# CRISPR-Cas Genome Editing Technique for Fish Disease Management: Current Study and Future Perspective

**DOI:** 10.3390/microorganisms10102012

**Published:** 2022-10-12

**Authors:** Md. Akib Ferdous, Sk Injamamul Islam, Nasim Habib, Mazen Almehmadi, Mamdouh Allahyani, Ahad Amer Alsaiari, Alaa Shafie

**Affiliations:** 1 Department of Fisheries and Marine Bioscience, Faculty of Biological Science and Technology, Jashore University of Science and Technology, Jashore 7408, Bangladesh; 2 The International Graduate Program of Veterinary Science and Technology (VST), Department of Veterinary Microbiology, Faculty of Veterinary Science and Technology, Chulalongkorn University, Bangkok 10330, Thailand; 3 Department of Clinical Laboratory Sciences, College of Applied Medical Sciences, Taif University, P.O. Box 11099, Taif 21944, Saudi Arabia

**Keywords:** CRISPR-Cas, fish, pathogens, phages, RNA

## Abstract

Scientists have discovered many ways to treat bacteria, viruses, and parasites in aquaculture; however, there is still an impossibility in finding a permanent solution for all types of diseases. In that case, the CRISPR-Cas genome-editing technique can be the potential solution to preventing diseases for aquaculture sustainability. CRISPR-Cas is cheaper, easier, and more precise than the other existing genome-editing technologies and can be used as a new disease treatment tool to solve the far-reaching challenges in aquaculture. This technique may now be employed in novel ways, such as modifying a single nucleotide base or tagging a location in the DNA with a fluorescent protein. This review paper provides an informative discussion on adopting CRISPR technology in aquaculture disease management. Starting with the basic knowledge of CRISPR technology and phages, this study highlights the development of RNA-guided immunity to combat the *Chilodonella* protozoan group and nervous necrosis virus (NNV) in marine finfish. Additionally, we highlight the immunological application of CRISPR-Cas against bacterial diseases in channel catfish and the white spot syndrome virus (WSSV) in shrimp. In addition, the review summarizes a synthesis of bioinformatics tools used for CRISPR-Cas sgRNA design, and acceptable solutions are discussed, considering the limitations.

## 1. Introduction

Fish diseases are a serious barrier in the aquaculture sector, affecting more than a billion dollars yearly. Climate change and developing fish farming may influence the balance or imbalance of pathogen, host, and environmental interaction, with new infections being detected or identified annually and more known diseases arising in various global regions and species [1]. Pathogen evolution is thought to be accelerated in intensive farming systems due to the high density of vulnerable hosts, which promotes pathogen transmission and virulence [2]. Because of higher population densities and host–pathogen interactions, this aspect of the farming environment is expected to extend to biological interactions between pathogenic bacteria and their phages, viruses, and parasites. However, many of these diseases or infections have no proven or approved recommended treatments, vaccinations, or control strategies and remain a substantial barrier to the economic sustainability of aquaculture in specific regions and species [1].

Aquaculture enterprises may need new scientific procedures to increase fish production while maintaining trait quality. Several initiatives have been conducted over the last two decades to manage and treat disease in aquaculture species, with varying degrees of success [3]. Many proven aquaculture species, such as tilapia, carp, salmonids, and some marine species (sea bass, sea bream, and grouper), have commercial vaccines for a limited number of diseases and authorized treatments for specific pathogens. However, there is significant variation from country to country and even within a geographic region [1]. Several diseases that have a substantial economic impact in aquaculture are viral infections with no therapies and vaccines, which, if produced, only provide limited protection. Numerous examples of bacterial, parasitic, and fungal diseases can pose significant international economic and welfare concerns to aquaculture. The management and control of parasitic infections are critical not only for the viability of the aquaculture sector but also for preventing horizontal parasite spread to wild fish [4,5]. Chemotherapy has had some promising results and has been proposed as a potential method of treating fish parasites [6]. Such initiatives, however, are incompatible with the United Nations’ Sustainable Development Goals, as well as generally agreed fish welfare and environmental standards. As a result, additional long-term preventative approaches for parasite management must be developed. On the other hand, in aquaculture, increasing production and the frequency of diseases in aquatic animals are driving up antimicrobial use and antimicrobial resistance [7,8] across various farmed aquatic species. Antimicrobial residues in the aquatic environment affect the environmental microbiome, affecting the ecosystem’s capacity for regulation, provisioning, and sustenance [9,10]. As a result, the enrichment of naturally existing pathogens in aquaculture habitats and the usage of antimicrobial treatments provide an appealing opportunity to investigate newer, more robust solutions for aquaculture sustainability.

Currently, biotechnology research can address several issues, not just related to aquaculture farming but also environmental issues [3]. Several genome-editing tools have recently been developed, including zinc-finger nucleases (ZFNs), transcription activator-like effector nucleases (TALENs), and more recently clustered regularly interspaced short palindromic repeats (CRISPR) and CRISPR-associated nucleases 9 (Cas9), which have made it possible to edit genes or knock out unwanted parts of them in various animal models [11]. The CRISPR-Cas9 system has been developed as a new elite genome engineering tool, even for organisms where genome editing would be challenging. With this promising new technology, it is possible to overcome several challenges that the aquaculture industry faces. Genomic editing using CRISPR can quickly introduce significant genome changes, making it useful for genetic improvements, disease resistance, and disease control in aquaculture [11,12]. For example, a novel mechanism for RNA-guided immunity against RNA viruses in vertebrates is provided by CRISPR-CasRx for engineering interference against RNA viruses in fish [13]. In addition, about half of all bacteria and almost all archaea possess a CRISPR-Cas system that protects them from foreign genetic elements, such as viruses and plasmids [14]. Thus, the role of the CRISPR-Cas mechanism in the aquaculture field would be crucial for future global food demand. There are several reviews on using CRISPR-based genome editing in aquaculture [15,16]; however, less effort has been made to apply CRISPR-Cas to control diseases in aquaculture. This review focuses on the pathogenic aspects of CRISPR-Cas9 genome editing relevant to aquaculture applications. Furthermore, a workflow for genomic interactions between CRISPR-Cas and phage is presented, primary techniques related to anti-RNA parasite experiments and outcome prediction of disease management in a CRISPR-Cas9 system are described, bioinformatics tools for CRISPR mechanisms are demonstrated, and the future of CRISPR-Cas9 for aquaculture disease management is briefly discussed in this study. We also mention aspects that need to be examined or improved for genome editing to effectively manage microbial diseases in fish farming. Finally, this study is intended to offer an overview of CRISPR genome-editing studies for fish disease management and treatment to encourage more genome editing research and uses in aquaculture.

## 2. Relationship between CRISPR-Cas and Phages

Bacteria-infecting viruses, generally known as bacteriophages, require a bacterial host to survive. Their numbers in the biosphere make them the most abundant [17]. Bacteriophages are a significant threat to bacteria due to their capacity to infect their bacterial host. When the host’s environment becomes unfavorable, phages may switch to a pseudolysogenic method. Pseudolysogeny is the development of bacteriophages in a host cell without multiplication or replication and occurs with zero degradation of the viral genome.

In such situations, prokaryotes, such as bacteria, adopt various defense mechanisms to protect themselves. CRISPR functions as a natural defense mechanism or adaptive immune system of prokaryotes against viral DNA, bacteriophages, and plasmids, which was first reported in the *E. coli* genome [18]. Adaptive immunity refers to the immunity that an organism acquires after exposure to an antigen, either from a pathogen or vaccine. It may be found in most lysogenic bacteria [19], including two aquaculture-related bacterial species, *Flavobacterium psychrophilum* [20] and *Vibrio anguillarum* [21]. The relationship between the phages’ life cycle and CRISPR-Cas is still poorly known [22].

**Figure 1:**
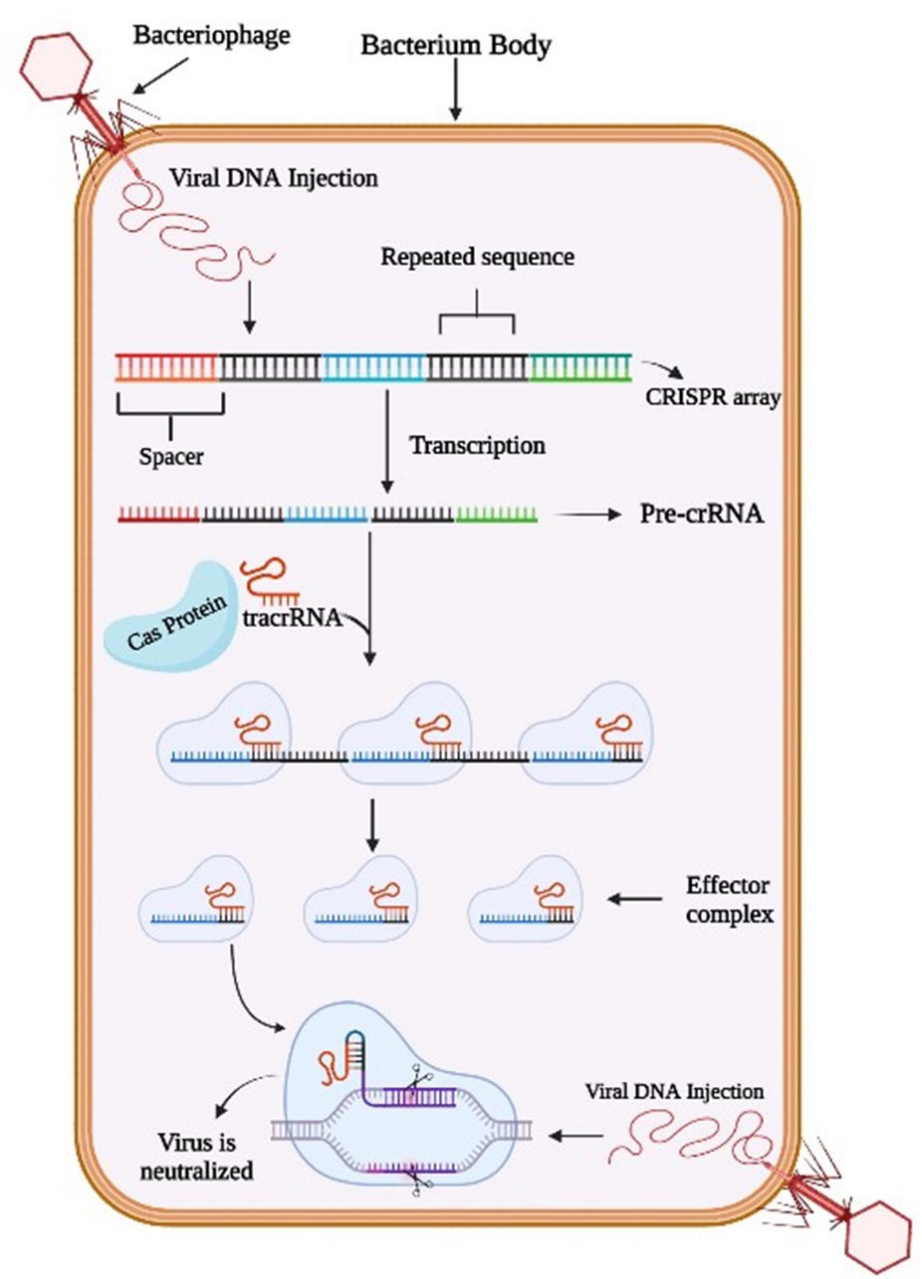
Mechanism of CRISPR-Cas against bacteriophage interference.

Regarding CRISPR, the repeated sequences of prokaryote DNA nucleotides are described as palindromic repeats because they are the same whether reading forwards or backward. The unique sequences nestled between the palindromic repeats are called spacers. Spacers are the DNA bits originating from the foreign mobile genetic elements (MGEs) that have previously infected the prokaryote and do not belong to the bacterium. Different spacers, potentially originating from different viruses, are sandwiched between the repeated sequences and produce a CRISPR array. In this way, bacteria retain a memory of a past infection [18]. The CRISPR array can undergo transcription to form CRISPR RNA (crRNA) called pre-crRNA. In the next step, the Cas protein becomes involved, which refers to the CRISPR-associate nuclease protein capable of cleaving DNA at specific nucleotide linkages. The presence of tracrRNA with Cas protein was also recorded. Each spacer and palindromic repeat end up with an effector complex consisting of a segment of pre-crRNA, a tracrRNA, and a Cas protein. By cleaving the strand between these complexes, the ribonuclease-3 enzyme helps the cell defend against the invader whose genome produced that crRNA. The whole process neutralizes the virus by preventing viral transcription [18]. The relation of the mechanism of CRISPR-Cas against bacteriophage interference is shown in Figure 1.

## 3. CRISPR-Cas for Anti-Parasitic Action

Invertebrate parasites may either be free-living or obligatory parasites that depend on their hosts for survival and reproduction. Both obligatory and opportunistic parasites may be found in fish, but obligatory parasites are mainly responsible for causing many parasitic infections in fish. Most fish that seem to be healthy often have small numbers of different parasites on or in their bodies, by which the fish usually suffer little to no danger. However, changes in water temperature or salinity reduce fish immunity causing a significant increase in the number of parasites per fish, and parasitic disease outbreaks frequently happen. In addition, there are connections between parasitic diseases and other infections. It has been noted that cultured fish in captivity are host to a wide range of parasites. Some of these parasites have led to severe disease outbreaks or persistent subclinical effects in farmed fish, costing fish farmers a lot of money. The fish are most vulnerable in the beginning phases of the culture cycle, especially when the fish are tiny and in the hatchery and nursery stages. The three major groups of parasitic organisms that infect farmed fish are protozoa, platyhelminthes, and crustaceans. Many of these parasites can potentially spread disease and result in significant financial losses.

Several therapies and preventative measures may be used to deal with parasite assaults, including environmental disinfection, seedling disinfection, health management, nutritional supplements, copper sulfate, potassium permanganate, formalin, zinc sulfate, and ivermectin. However, the use of medications and antibiotics harms the environment and the safety of food. From this perspective, CRISPR-Cas may be a more efficient way to create a species-specific insecticide for defeating parasite infestations by a genetic operation that targets a particular place of the gene for cutting and repair. The most often researched fish parasite species of the *Chilodonella* protozoan group, *Chilodonella piscicola*, *Cryptocaryon irritans*, and *Chilodonella uncinata*, have all been subjected to CRISPR-Cas anti-parasitic activities [23,24,25]. These hypothermic protozoan parasite species oversee, causing both gill and skin diseases in freshwater fish, which restrict the development of both juvenile and adult fish, especially in spring and fall [25].

Yige Li et al. reduced the survival capacity of *C. piscicola* by destroying the parasite DNA at a particular location using a combination of Cas9 messenger RNA (mRNA) and single guide RNAs (sgRNA) [25]. In that experiment, the CRISPR-Cas9 system was used to design sgRNA in conjunction with the known sequences of the 18S ribosomal RNA (rRNA), internal transcribed spacer 1 (ITS-1), and 5.8S ribosomal RNA (rRNA) of *C. piscicola* to destroy the genetic barcode of *C. piscicola*. As evidence of the efficiency of the chosen sgRNA of 18S rRNA sequence and ITS-1 sequence, the survival rate of the experimental *C. piscicola* was decreased to 40% compared to the blank control group with zero significant difference. It demonstrated that both sites had the potential to eradicate *C. piscicola* successfully. According to real-time quantitative PCR (RT-qPCR), Cas9 may either act with a single sgRNA or a combination of two sgRNAs to damage the parasite DNA in a particular area [25].

**Figure 2:**
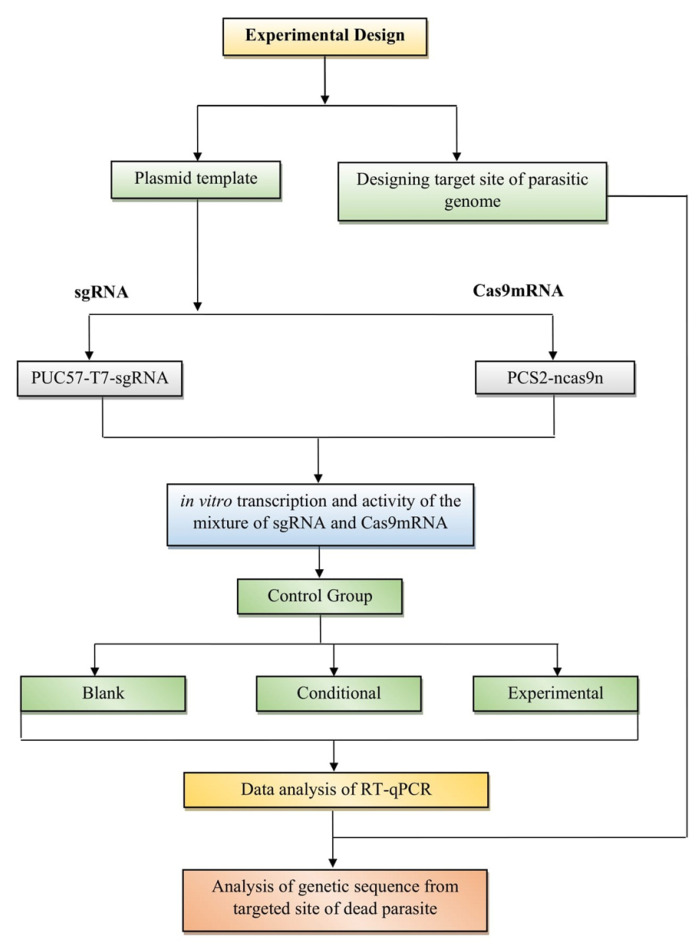
Experimental design of developing an RNA anti-parasite.

Majeed et al. also conducted a similar study to control the *Aphanomyces invadans* pathogen, a causative agent for epizootic ulcerative syndrome (EUS) [26]. In that experiment, scientists applied the CRISPR-Cas9 system for editing the *A. invadans* genome by targeting the serine protease gene in in vitro and also observed the effect on the virulence and pathogenicity of the *A. invadans* in vivo. They designed three single guide-RNAs (sgRNA) combined with the Cas9 to form a ribonucleoprotein (RNP) complex and transfected in *A. invadans* protoplasts and zoospores. Three groups of dwarf gourami *(Trichogaster lalius)* were taken as test species and experimentally inoculated with (i) non-treated zoospores; (ii) RNP-treated zoospores; and (iii) autoclaved pond water as a negative control to investigate the effect on the virulence in vivo. According to the in vivo results of the study, the CRISPR-Cas9-treated *A. invadans* zoospores did not express any signs of EUS in the fish [26]. The basic experimental design of developing an RNA anti-parasite using the CRISPR-Cas method is shown in Figure 2.

## 4. CRISPR-Cas for Developing RNA-Guided Immunity against RNA Viruses in Fish

One of the deadliest viruses that may infect fish is the RNA virus, which is unpredictable and challenging to prevent. They are highly dynamic pathogens because of their short generation times, enormous population numbers, and high mutation rates, among other distinctive traits. Iridoviridae, Adenoviridae, and Herpesvirdae are home to fish viruses with DNA genomes. In contrast, those with RNA genomes are found in the families Picornaviridae, Birnaviridae, Reoviridae, Rhabdoviridae, Orthomyxoviridae, Paramyxoviridae, Caliciviridae, Togaviridae, Nodaviridae, and Retroviridae.

RNA-guided immunity against RNA viruses could be developed in the fish body using the antiviral CRISPR-Cas, which had previously been successfully targeted by CRISPR-Cas13 [27,28,29]. On the other hand, CasRx, a small type VI-D effector (Cas13d), can also effectively knock down RNA in RNA viruses [13]. The RNA-targeting CRISPR-CasRx is a programmable system, and Cas13d is a CRISPR effector known as small type VI.

The two primary categories of CRISPR-Cas systems are class I, which mediates the interference via multi-effector complexes, and class II, which utilizes a unified, multi-domain effector [30]. In addition, these classes are further divided into six types and thirty-three subtypes based on the genomic architecture of the CRISPR array and its distinct interference effectors. Types II, V, and VI of the CRISPR-Cas systems are within class II. Endonucleases of types II and V are used for the DNA, while those of type VI are only used for RNA [30,31]. To provide prokaryotes with protection against RNA, class II type VI CRISPR-Cas systems use an RNA-guided and RNA-targeting mechanism [32]. The Cas13 effector protein is the same for all type VI CRISPR-Cas systems. Multiple studies have shown further variations of Cas13 proteins belonging to various Cas13 families, which have been categorized into four type VI subtypes (subtypes A–D) [28,30,33]. A novel Cas13 subtype known as CRISPR-Cas13d (CasRx) has also recently been discovered by researchers; it has minimal sequence similarity with earlier Cas13 effectors [33,34]. Compared to other Cas13 effectors, CasRx is more efficient and more robustly activated in cells when RNA-guided RNA cleavage occurs [33,34]. It provides the most effective targeting and has the smallest size, making it perfect for in vivo therapeutic applications [34].

**Figure 3:**
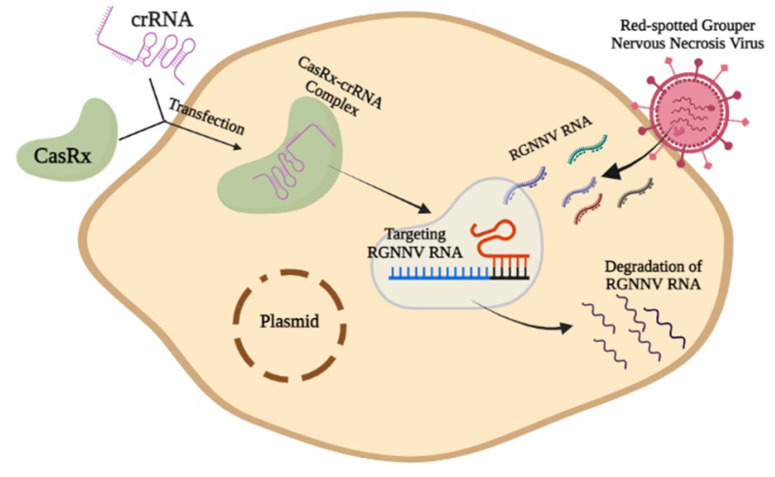
The in vitro RGNNV targeting via the CasRx system.

Qing Wang et al. designed synthetic mRNA coding for CasRx and used CRISPR RNAs to guide it to target the nervous necrosis virus (NNV), which is an RNA virus of fish [35,36], and applied the coding both in vitro and in vivo to observe the significance of the dominating RNA virus [13]. Scientists selected the red-spotted grouper (*Epinephelus coioides*) as a model species for the experiment. The red-spotted grouper NNV (RGNNV) is one of the four classified NNVs; striped jack NNV, tiger puffer NNV, and barfin flounder NNV are the remainder of them [36,37]. NNV is also found in orange-spotted grouper, Sevenband grouper, brown-marbled grouper, turbot, etc. Nervous necrosis viruses (NNV) are icosahedral non-enveloped single-stranded positive-sense RNA viruses (ssRNA+ viruses) classified in the family *Nodaviridae* which is the pathogen of viral nervous necrosis disease (VNN) that destroys the central nervous system of infected fish [38,39]. The in vitro RGNNV targeting via the CasRx system is shown in Figure 3.

Scientists designed the experiment in three basic steps. Firstly, to increase the protein content of CasRx, researchers improved the codons that express it. Secondly, to enhance crRNA expression, the zebrafish U6 promoter was applied. Thirdly, they designed CasRx to eliminate the differences in cellular location.

RGNNV is composed of CP and RdRp (Clamping RNA-dependent RNA polymerase). They investigated the possibility that CasRx may target the RGNNV CP and RdRp to induce effective and reliable RNA virus interference [13]. In this research, scientists developed three crRNAs for targeting the sequences of code of CP mRNA and two crRNAs for targeting the sequences of code of RdRp mRNA. To investigate the effect of the CasRx-crRNA complex on the cellular level of RGNNV, grouper spleen (GS) cells were treated with plasmids carrying CasRx and crRNA. The first CasRx expression was observed after 6 h of transfection, and it became more expressive after 12 and 24 h, but it did not show much expression between 24 and 48 h. For each of the five crRNAs, RGNNV infection was carried out after transfecting GS cells with plasmids carrying CasRx-dNLS or CasRx-NLS. All ten combinations reduced the number of viral RNA copies. Furthermore, the virus titer data showed that transfection of cells with CasRx-dNLS or CasRx-NLS and crRNA significantly reduced viral titers and RGNNV pathogenicity. Extensive CPEs were shown when GS cells were treated with RGNNV or CasRx plus nonspecific (ns)-crRNA and RGNNV. Few CPEs were seen when cells were transfected with either CasRx-dNLS or CasRx-NLS and each crRNA. Additionally, the findings of the immunofluorescence experiment showed that GS cells subjected to only RGNNV or CasRx plus ns-crRNA and RGNNV displayed strong fluorescence signals of the RGNNV CP protein. Positive fluorescence signals were markedly reduced when cells were transfected with either CasRx-dNLS or CasRx-NLS and each crRNA. These findings demonstrated that the CasRx system effectively prevented RGNNV infections in vitro [13].

The effectiveness of CRISPR-CasRx has been proven to combat an RNA virus in vertebrates. The degradation of several viral genomic areas led to decreased CP and RdRp mRNA levels, in vitro cell vacuolation, and cumulative mortality in vivo. These results demonstrated the efficacy of using CRISPR-CasRx, a vertebrate RNA virus that may be targeted and interfered with by reducing RGNNV replication and dissemination.

## 5. Application of CRISPR-Cas in Fish Disease

Aquaculture industries worldwide face serious problems such as infectious and parasitic diseases, reduced viability, decreased fertility, poor development, environmental contamination by escapee fish, coastal conflicts, and disagreements over the patenting of research products [15,40]. Among these, disease outbreaks in aquaculture are a major issue that are one of the main reasons for the reduction in fish production. Many reputable shellfish farms report shellfish dying overnight because of viral assaults. In fish aquaculture, reproduction, and development [41,42], growth [43], pigment [44,45], disease resistance [46], trans-GFP usage in research [47], and omega-3 metabolism [48,49] are the qualities that are most often targeted for genetic engineering [50]. However, using molecular biological techniques to resolve diseases has become a core technology. Genomic editing (GE) has created several controls for aquatic diseases, and it will continue to do so in the future in a variety of different ways. Among these GE methods, CRISPR-Cas has been applied to modify several genes for targeting species-specific pathogens as modern technology.

CRISPR-Cas has been applied in immunological studies in channel catfish (*Ictalurus punctatus*) according to several types of research [51,52]. It enhanced the resistance of channel catfish to many diseases by injecting the alligator cathelicidin gene into the fish [53]. Additionally, this technology enhances the fish body’s natural immunity, which works against bacterial diseases or other infectious diseases such as *Edwardsiella ictalurid* and *Flavobacterium columnare* [54]. The editing of disease-resistance genes in channel catfish is an additional application of CRISPR-Cas of commercial relevance [51,52,55].

In shrimp and prawns, the eyestalk neuroendocrine complex contains suppressing/inhibiting substances that always prevent breeding and spawning under captivity. These limiting elements also hinder the process of growth. These aquatic organisms’ immune systems have reportedly been weak, making viral and bacterial diseases highly likely to strike them. Certain marine shrimps have already had their gonad-inhibiting hormone (GIH) and molt-inhibiting hormone (MIH) genes evaluated [56,57,58]. Using CRISPR-Cas technology has been able to eliminate the harmful effects of hormones on growth and reproduction, which may open the way to developing a powerful substitute for eyestalk ablation that has a comparable effect. Some researchers have tried to delete the gene using this RNA interference method [59,60]. When working on *Penaeus monodon* (giant tiger prawn), Treerattrakool et al. used the method of RNA interference to induce maturity in both wild and captive shrimp and reported that shrimps injected with anti-GIH double-stranded (ds) RNA showed enhanced maturation [59]. According to Das et al., RNA interference was used to silence the gonad-inhibiting hormone gene in the eyestalk neuroendocrine complex of the *P. monodon* (tiger shrimp) [60]. They discovered a three–five times increase in the transcript of the androgenic gland hormone (AGH) in males but no alteration in the expression of vitellogenin in females. Additionally, CRISPR-Cas technology can be utilized to manage bacterial and viral infections, particularly in shrimp and prawns. The CRISPR-Cas process in shrimp and prawns may also function similarly to that of bacteria when viral DNA attacks them. For example, CRISPR-Cas can replicate and insert portions of the white spot syndrome virus (WSSV) DNA into shrimp genomes as “spacers” between the short DNA repeats in CRISPR when WSSV invades them. By providing a template for RNA molecules to rapidly recognize and target the same DNA sequence in the case of future viral infections, these spacers improve the immune response of shrimp. The RNA molecules redirect the CRISPR complex to an incoming sequence of foreign DNA if they recognize it. There, the Plasmid Cas proteins of the shrimp cut the invading gene and render it inactive. The shrimp may be shielded against contagious infections because of this [60].

Culturing commercial species in the aquatic environment, every year, significant losses are attributed to mass mortality, rejection of aquaculture species’ shipments due to a lack of quality standards, the impact of biotic and abiotic stresses on aquaculture species, and the absence of standardized disease control and pollution-impact methods or protocols [3]. However, at this point, we require highly potent technologies to solve some of the significant problems in the aquaculture sector. With the development of CRISPR-Cas technology, it may be possible to solve any biological problems relating to genetic diseases or other problems without significantly changing the genetic makeup of aquaculture species and preventing viral and bacterial infections; CRISPR-Cas technology can prove to be a potent tool [3].

## 6. Advances in Bioinformatics in CRISPR-Cas

Bioinformatics is a scientific field that generates methodologies and software tools for analyzing biological data. It has been applied in various applications such as in silico studies of biological questions utilizing computational and statistical tools. It is frequently used to find potential genes and single nucleotide polymorphisms (SNPs). Furthermore, a field of study known as proteomics in bioinformatics seeks to comprehend the organizing concepts found in nucleic acid and protein sequences [61]. The main effects of bioinformatics have been the automation of microbial genome sequencing, the creation of integrated databases accessible through the internet, and genome analysis to comprehend gene and genome function. Bioinformatics is now used for a wide variety of other significant tasks in addition to the analysis of gene variation and expression, the analysis and prediction of gene and protein structure, as well as the prediction and detection of gene regulatory networks. It can analyze data more quickly to enhance the accuracy of the findings and explain the causes and phenomena of diseases at the gene/pathway level.

The first thing we must understand before relating the CRISPR-Cas system to bioinformatics is that selecting the appropriate CRISPR target gene is an essential step in successfully targeting gene editing. Bioinformatics can be used to locate and insert CRISPR-Cas into the targeted genome [3]. The choice of the target site is constrained by the possibility of off-target editing and variations in editing effectiveness. Numerous computational techniques have been created in recent years to assist researchers in choosing target sites for CRISPR knock-in/out experiments. In developing single-guide RNA (sgRNA) for CRISPR applications, these methods are likely to be helpful in both target site selection and sgRNA creation. The sgRNA design tools are specifically suitable for genetic screening and CRISPR-mediated gene regulation research has also been developed, resulting from the expansion of CRISPR applications. Computational tools have been created to analyze CRISPR genome-edited data produced by Next Generation Sequencing (NGS) systems and aid in sgRNA creation [62]. The CRISPR-Cas9 genome-editing technologies use programmable nucleases to accurately and frequently modify a particular section of the genome which may use RNA-guided nucleases [63,64,65].

CRISPR-Cas has successfully modified specific genomes in significant model species, such as zebrafish [66]. It modifies two RNAs—a transactivating CRISPR RNA (tracrRNA) that base pairs with the crRNA and a CRISPR RNA (crRNA) complementary to the targeted DNA sequence—that recruit Cas9 to the target site. The target sequence should be followed by a protospacer adjacent motif (PAM) sequence for recognition (nGG, where n can be any nucleotide). The crRNA and tracrRNA may be combined to form a single synthetic guide RNA (sgRNA) [66] that works efficiently with Cas9 to cause cleavage of the target site (~20 bp), which must come after the PAM sequence in the genome. Using in vitro transcription promoters such as T7, T3, or SP6 to create sgRNAs restricts the target sequence. Here, the CRISPR-Cas system was used to modify the *Xenopus tropicalis* (western clawed frog) genome, providing another tool for quick and effective targeted mutagenesis.

Cas 9 is a CRISPR-related protein adapted from a naturally occurring genome-editing system and used here as a bacterial immune defense. Most genomic restriction nucleases require substantial and complex PAM sequences that would restrict them due to reduced genome size. Distinct PAMs in the SpCas9 system are used for genome manipulation, including target gene disruption and single base-pair mutations in various organisms and cells. Developing the SpCas9 to identify more PAMs would be an alternative approach to increasing PAM specificity. Although SpCas9 is the most well-known nuclease, Cas9 can also be obtained from many bacterial species. The fundamental difference between them is the PAM sequence required for the cleavage of Cas9 nucleases from different bacteria [67].

The CRISPR-mediated genome editing tools are shown in Table 1, which are used for fish.

On the other hand, many genetic disorders and undesirable features are carried on by base-pair changes in the genomic DNA. Base editing, the most recent development of CRISPR-Cas-based technologies, may directly introduce point mutations into cellular DNA without leading to a double-strand DNA break (DSB). The CRISPR-base-edit tools have recently increased by prime editing (PE), which now includes all twelve potential transition and transversion mutations in addition to minor insertion or deletion changes [77]. The base editing resources are shown in Table 2.

In addition, to targeting single-point mutations, CRISPR has provided a variety of methods for editing the genome precisely, such as functional gene knockouts and epigenome modifications. Recent research has focused on improving Cas9 selectivity and expanding target coverage; guided evolution has resulted in discovering many Cas9 variants that will significantly expand targeting coverage. Base-editing techniques have also made significant advancements in the investigation of pathogenic variations in animal models; they will speed up the functional verification of potential disease genes in model organisms and the creation of therapeutic tools for the treatment of several disorders [83]. The design of efficient sgRNAs is becoming increasingly difficult since the CRISPR-Cas9 system has swiftly become a ubiquitous gene-editing tool in biological research. To address this critical issue, various bioinformatics techniques have been created. In conclusion, by enhancing experimental planning, data integrity, and computational modeling, researchers created a novel sgRNA design tool that consistently outperformed in various experimental conditions [84].

## 7. Limitations of CRISPR-Cas for Aquatic Disease Perspective

The field of molecular biology is being revolutionized by the quick advancement of genome-editing technology such as CRISPR-Cas, which allows DNA modification in a broad range of species. It is being considered for several applications, from agriculture to clinical therapeutics [85]. CRISPR-Cas technology has made tremendous strides in recent years and demonstrated significant promise in several areas of life sciences’ study.

Despite the impressive CRISPR advancements, a few issues still need to be resolved to develop Cas systems to their full potential. CRISPR technologies have some primary limitations as application measures and genetic perspectives. These technical difficulties can be resolved in the present attempts to address all of those worries.

The CRISPR-Cas method is a relatively new gene-editing technology; generally, the methods associated with gene editing are quite expensive. Such an expensive technology in the molecular biological sector, such as CRISPR-Cas, is challenging to adapt in a developing or underdeveloped agriculturally dominant country. As a result, it is tough to quickly implement this technology in aquaculture in these countries, even if it is effective enough to control fish diseases. In addition to being expensive, this technology is also quite complicated. These complexities make it difficult to implement CRISPR-Cas as a commercial method. As a result, institutional education on this method is primarily essential. On the other hand, not all laboratories have sufficient equipment to run this technology except specialized and facilitated laboratories. Due to the problem of inadequate equipment, it is impossible to conduct this technology in all institutional laboratories.

Moreover, from a genetic perspective, the insufficient aquatic genomic resource is the major limitation of CRISPR technology. Although scientists have vast genetic information about some worldwide, commercially important model species (e.g., *Nile Tilapia*, *Atlantic Salmon*), they are insufficient compared to the total number of aquaculture species, which is over 600 according to the FAO [86]. Furthermore, the identification of trait-related genes is required for the genetic functioning of aquatic species to locate which gene should be targeted [86]. On the other hand, the problem of genomic duplication in fish is more than in other aquatic organisms [87]. In addition, another significant issue with using CRISPR-Cas in treating fish disease is off-target mutations at unwanted sites other than the desired on-target sites [88].

There may have some possible solutions to overcome these limitations. First, future aquatic genome-sequencing will be aided by the reduced cost of sequencing, allowing for establishing the essential genetic base. Second, the steps to start introductory institutional courses on CRISPR-Cas can be helpful in the skill development and enhancement of lab facilities. For the solutions of genetic perspectives, increasing refinements in QTL methods will result in more trait-related genes being identified [89]. On the other hand, potential candidates should be targeted for genes that impart advantageous features across species and lines. A well-designed annealing sgRNA may either avoid or identify off-target mutations by comparing it to current genome assemblies [50].

## 8. Future Perspective and Approaches

Although CRISPR-Cas technology is relatively new, it can be undoubtedly said that scientists will rely heavily on it in the future to diagnose and prevent fish diseases. With time, the versatile application of this technology is turning it into a multi-dimensional technology. It is possible to paint an imaginary picture of the future perspective and approaches of CRISPR-Cas. In this step, an attempt can be made to assess this technology’s future status and needs, based on several aspects of fish disease diagnosis and health management.

- Antibiotics’ massive selection pressures brought on by antibiotic exposure cause commensal and pathogenic microorganisms to develop and propagate antibiotic resistance. This strategy is paradoxical for preventing the fast evolution of new antibiotic-resistant organisms because of the lengthy process of discovering new antibiotics. To deal with diseases brought on by resistant superbugs, alternative strategies including creating nucleic acid-based anti-bacterial therapeutics, anti-bacterial peptides, bacteriocins, anti-virulence chemicals, and bacteriophage therapies should be used. To address antibiotic resistance in this situation, scientists have already begun to use the recently popular CRISPR-Cas system [90]. Antibiotic-resistant superbugs are one of the major concerns today, but CRISPR technology is expected to protect from this problem if used properly;
- Antibiotics target cellular processes or activities, such as nucleic acid synthesis and cell membrane formation, to impact specialized bacterial mechanisms. These processes cannot destroy specific pathogens in the diverse microbial community—antibiotics damage both the members of the beneficial microbiota and the bacteria that cause infections. There is currently no antibiotic method that targets exclusively pathogenic bacteria. The use of antibiotics nowadays is not species-specific. The CRISPR-Cas9 gene-editing technique and its applications against bacteria will be a crucial strategy to stop the clonal proliferation of dangerous bacteria, offering a novel remedy to the world-wide issue [91];
- Among all of the pathogens that cause disease in the fish body, viral diseases can be considered the most dangerous. In particular, fish diseases by RNA viruses cause the most suffering to scientists and fish farmers. From that point of view, since RNA viruses show mutations or Single Nucleotide Polymorphisms (SNP) so frequently, any preventive measures designed to target a particular virus may no longer work after the mutation or SNP. The CRISPR-Cas method can play a vital role in solving this problem in the future. CRISPR-Cas technology has already experimented with the RNA virus targeting method for red-spotted grouper nervous necrosis virus (RGNNV) in fish [13]. Scientists found success in this experiment by using the CasRx-crRNA complex;
- Apart from these, CRISPR has also been applied for anti-parasitic action. Scientists are also succeeding in this area [25]. Moreover, using this technology, genetically improved or modified species can be created that will be born with high immunity from the beginning of life.

## 9. Conclusions

Aquaculture offers microbial populations semi-natural and generally ideal environments. For this reason, pathogenic attacks in aquaculture are always a significant issue that have a direct and adverse effect on production, as the fish are considered an easy host for causative agents. Among all preventive measures, we have focused on the CRISPR-Cas method in this review paper because of the popularity and dependable image created by the vast research interests and practice of CRISPR-Cas in disease management sectors. The CRISPR-Cas system has improved genome-editing technology and shown significant promise in controlling aquaculture diseases. We have reviewed the recent research and projected the uses of CRISPR-Cas in the aquaculture industry. We have discussed the case studies that have previously been conducted on the use of CRISPR-Cas for creating anti-parasitic RNA, as well as the advancement of the CasRx-crRNA complex against fish RNA-virus. Although the use of CRISPR-Cas in aquaculture disease research is still in its early stages compared to its usage in biomedical research, we have reviewed its limits and applications as a new method and attempted to relate it with bioinformatics.

## Figures and Tables

**Table 1:** CRISPR-Cas-related genome editing resources that may be appropriate for use in aquaculture.

Name	Function	URL	Reference
CRISPRScan	CRISPRscan is used to evaluate every Cas9 sgRNA. Generate Cas9/Cas12a targets	https://www.crisprscan.org accessed on 1 September 2022	[68]
CHOPCHOP	Tool to design TALEN mode, CRISPR-Cas9, CRISPR-Cas9 Nickase, CRISPR-Cpf1, or TALEN mode.	http://chopchop.cbu.uib.no accessed on 1 September 2022	[69]
ccTop	Tools for target selection and off-target prediction for multiple Cas9 and Cas12a.	https://crispr.cos.uni-heidelberg.de accessed on 1 September 2022	[70]
Cas-Designer	Cas9, Cas9 variations, and Cas12a targets can be designed with this tool.	http://www.rgenome.net accessed on 1 September 2022	[71]
MENTHU	It is a tool for locating DNA sites likely to result in Predominant MMEJ Allele (PreMA) double-strand DNA break repair.	http://genesculpt.org/menthu accessed on 1 September 2022	[72]
CRISPR-ERA	Tool for genome editing as Cas9 nuclease. Create sgRNAs for gene activation or repression	http://crispr-era.stanford.edu accessed on 1 September 2022	[73]
CRISPResso 2	Analysis of the results of genome editing from deep sequencing data	http://crispresso.pinellolab.partners.org accessed on 1 September 2022	[74]
Cas-Analyzer	High-throughput sequencing data collection tool for genome-edited cells	http://www.rgenome.net/Cas-analyzer accessed on 2 September 2022	[75]
CRISPR-GA	Used to find indels in data from next-generation sequencing.	http://crispr-ga.net accessed on 2 September 2022	[76]
CRISPRz	Collection of verified CRISPR targets in zebrafish	https://research.nhgri.nih.gov/CRISPRz accessed on 2 September 2022	[77]
inDelphi	Tool for predicting indels caused by microhomology-mediated end-joining (MMEJ) and non-homologous end-joining (NHEJ) repair.	https://indelphi.giffordlab.mit.edu accessed on 2 September 2022	[78]
FORECasT	Tool for predicting the mutational effects of double-stranded breaks generated by CRISPR-Cas9.	https://partslab.sanger.ac.uk/FORECasT accessed on 2 September 2022	[79]

**Table 2:** Base editing resources.

Resources	Function	URL	References
BE-Analyzer	Used as a rapid evaluation tool for CRISPR-base edited cells of NGS data.	http://www.rgenome.net/be-analyzer accessed on 2 September 2022	[80]
BE-Designer	Used for CRISPR base editing, a designer of guide RNA.	http://www.rgenome.net/be-designer accessed on 2 September 2022	[80]
BEEP	Used for analysis of Sanger sequencing ab1 files for CRISPR-mediated base editing effectiveness.	https://github.com/mitmedialab/BEEP accessed on 3 September 2022	[81]
CRISPR-SKIP	Used to select the exons that can be skipped by modifying the flanking G nucleotide.	https://knoweng-0.igb.illinois.edu/crispr-skip accessed on 3 September 2022	[82]
CRISPResso 2	Used as a tool for next-generation sequencing data.	http://crispresso.pinellolab.partners.org accessed on 3 September 2022	[74]
EditR	A single Sanger sequencing run can be used to predict possible editing in a guide RNA region.	http://baseeditr.com accessed on 3 September 2022	[83]
iSTOP	A database of sgRNAs for CRISPR-dependent base editing of STOP codons (sgSTOPs).	https://www.ciccialab-database.com accessed on 3 September 2022	[84]
Beditor	Designing Guide RNA Libraries for CRISPR-Mediated Base Editing	https://github.com/rraadd88/beditor accessed on 3 September 2022	[85]

## Data Availability

Not applicable.
